# Dr. C. C. Blackburn

**Published:** 1889-05

**Authors:** 


DR. C. C. BLACKBURN, late editor of The *Madisonian* has again become a citizen of Atlanta. Dr. B. is an old and somewhat distinguished member of the profession. Once a professor in the Oglethorpe medical school at Savannah, and associate editor of the *Oglethorpe Medical Journal*, he has also been engaged in editorial labors in other departments. The Doctor combines a taste for medicine, politics and literature, and in the several localities in Georgia, in which he resided, he made himself known and felt as an active and progressive man. We welcome him back to Atlanta in which we trust he will find a wider field for usefulness and success.

